# Endurance and Resistance Training Affect High Fat Diet-Induced Increase of Ceramides, Inflammasome Expression, and Systemic Inflammation in Mice

**DOI:** 10.1155/2016/4536470

**Published:** 2015-12-14

**Authors:** Cornelia Mardare, Karsten Krüger, Gerhard Liebisch, Michael Seimetz, Aline Couturier, Robert Ringseis, Jochen Wilhelm, Norbert Weissmann, Klaus Eder, Frank-Christoph Mooren

**Affiliations:** ^1^Department of Sports Medicine, Institute of Sports Sciences, Justus-Liebig-University, 35394 Giessen, Germany; ^2^Institute of Clinical Chemistry and Laboratory Medicine, University of Regensburg, 93053 Regensburg, Germany; ^3^Universities of Giessen and Marburg Lung Center, German Center for Lung Research, Excellence Cluster Cardio-Pulmonary System, Justus-Liebig-University, 35392 Giessen, Germany; ^4^Institute of Animal Nutrition and Nutrition Physiology, Justus-Liebig-University, 35392 Giessen, Germany

## Abstract

The study aimed to investigate the effects of differentiated exercise regimes on high fat-induced metabolic and inflammatory pathways. Mice were fed a standard diet (ST) or a high fat diet (HFD) and subjected to regular endurance training (ET) or resistance training (RT). After 10 weeks body weight, glucose tolerance, fatty acids (FAs), circulating ceramides, cytokines, and immunological mediators were determined. The HFD induced a significant increase in body weight and a disturbed glucose tolerance (*p* < 0.05). An increase of plasma FA, ceramides, and inflammatory mediators in adipose tissue and serum was found (*p* < 0.05). Both endurance and resistance training decreased body weight (*p* < 0.05) and reduced serum ceramides (*p* < 0.005). While RT attenuated the increase of NLRP-3 (RT) expression in adipose tissue, ET was effective in reducing TNF-*α* and IL-18 expression. Furthermore, ET reduced levels of MIP-1*γ*, while RT decreased levels of IL-18, MIP-1*γ*, Timp-1, and CD40 in serum (*p* < 0.001), respectively. Although both exercise regimes improved glucose tolerance (*p* < 0.001), ET was more effective than RT. These results suggest that exercise improves HFD-induced complications possibly through a reduction of ceramides, the reduction of inflammasome activation in adipose tissues, and a systemic downregulation of inflammatory cytokines.

## 1. Introduction

Obesity is often associated with a systemic low grade inflammation [1]. One of the prominent hallmarks of obesity-induced inflammation is a shift in the phenotype of macrophage and T-cell populations present in adipose tissue (AT) reflecting in a unique set of pro- and anti-inflammatory cytokines [2]. Free fatty acids and saturated fatty acids (SFA) are suggested to induce inflammation by activation of Toll-like receptors (TLRs) 4 and 2 in adipose tissue and macrophages, culminating in an increased activation of nuclear factor kappa-B (NF-*κ*B) thus increasing the expression in proinflammatory cytokines like TNF-*α* or IL-6 [2, 3]. Apart from SFA, a deregulated ceramide synthesis has also been suggested to play an important role in obesity related inflammation [4, 5].

Aerobic exercise is known to have triglyceride-lowering effects accompanied by a reduction of visceral and abdominal adipose tissue in overweight and obese subjects [6, 7]. These metabolic effects of exercise training are regularly accompanied by a reduction of the inflammatory status [8, 9]. Thereby, exercise training was followed by a reduced expression of both TLRs and several proinflammatory cytokines [9, 10]. However, most studies on exercise effects on obesity investigated the effect of aerobic/endurance training [3, 11]. Resistance training received less attention, but there are several evidences that it also affects metabolic disorders and inflammation in obesity [12–16].

Insulin resistance is a common complication of obesity-induced inflammation and the reduction of blood glucose a desirable outcome of weight loss programs [17]. In recent years, the nucleotide-binding domain and leucine-rich-repeat and pyrin domain containing 3 (NLRP-3) inflammasome has been identified as a major contributor to the development of systemic inflammation and insulin resistance [18]. NLRP-3 assembly is initiated upon TLR activation, extracellular ATP, or active interleukin 1*β* (IL-1*β*). Expression of proIL-1*β* and proIL-18 is NF-*κ*B dependent which in turn depends on activation through TNF-*α*. The maturation of NLRP-3 renders caspase 1 active that cleaves inactive proIL-1*β* and proIL-18 into their active forms which are then secreted into the surrounding tissue [18].

It was also found that ceramides induced the NLRP-3 inflammasome, driving the secretion of IL-1*β* followed by the development of insulin resistance [18]. Ceramides induce insulin resistance through the dephosphorylation of serine/threonine kinase Akt/PKB, hence disabling the translocation of GLUT4 to the cell membrane and reducing the uptake of glucose [19–21]. Aerobic exercise reduced ceramides in humans and ceramidase activity in rat skeletal muscle [22]. However, the effects of differentiated exercise regimes on ceramide levels, AT inflammation, and systemic cytokine levels in mice have so far not been investigated in one cohesive study. Therefore, we investigated whether different exercise regimes affect glucose tolerance, serum fatty acids and ceramides, inflammatory parameters in AT, and several inflammatory cytokines in serum of HFD fed mice. We hypothesized that both types of exercise are effective in reducing glucose intolerance. Moreover, these effects might be achieved most likely via modifying serum ceramide levels as well as local and systemic inflammatory markers. Endurance and resistance training differ in their muscle fiber response. Whereas endurance training addresses mainly type I and type IIA muscle fibers, resistance training is followed by a hypertrophy of types II and IIX [23, 24]. Finally, it is suggested that endurance and resistance exercise might address different targets of these pathophysiological signaling cascades. Therefore, a differentiated effect of endurance and resistance training would be expected.

## 2. Material and Methods

### 2.1. Animals, Diets, and Experimental Design

Male C57BL/6 mice (*n* = 36), 10 weeks of age, were kept at 21 ± 1°C in a reverse dark-light cycle (light on from 9 pm to 9 am). Mice were randomly assigned to 6 groups (5 to 8 mice per group): standard chow/sedentary (ST/S, Altromin 1320, Altromin Spezialfutter GmbH & Co., Germany), standard chow/endurance training (ST/ET), standard chow/resistance training (ST/RT), high fat/sedentary (HFD/S, TD 03584, Harlan Teklad, Germany), high fat/endurance training (HFD/ET), and high fat/resistance training (HFD/RT). The assigned diet was fed for 4 weeks before exercise protocols were implemented. Briefly, the ST diet contained 3.4% lipids and the HFD 35% by weight. A more detailed specification of the fatty acid composition can be found in Table 1. Mice were caged separately, with free* ad libitum* access to food and water. The animals were put on their diet 4 weeks before the 10-week exercise protocols commenced, which means a total of 14 weeks on the diet. Weight was recorded before and after 14 weeks on the diet. The animals were handled according to the Guide for the Care and Use of Laboratory Animals. The experimental design was approved by the Animal Welfare Officer of the Justus-Liebig-University and the regional council of the city of Giessen (number 94/2010).

### 2.2. Determination of Endurance Capacity and Exercise Protocols

Before ET commenced, VO_2_max was determined for each individual animal as described previously [25]. Eighty percent VO_2_max was used as threshold for the following exercise protocol. Each mouse assigned to the endurance group was trained on an electrical treadmill (custom-made) at 80% VO_2_max for 30 min/day, 5 times/week for 10 weeks. Briefly, by using a treadmill spiroergometry (custom-made), maximal oxygen uptake (VO_2_max) and maximal running speed (Vmax) of mice were determined. The treadmill was placed in a metabolic chamber where air was led through at a rate of 0.5 L/min. Samples of 200 mL/min of gas were extracted to the paramagnetic oxygen analyzer (type 1155, Servomex) and the carbon dioxide analyzer (Lair 12, M&C Instrument). All animals were acclimated to the treadmill before VO_2_max and Vmax were tested during a continuous, progressive test on the treadmill ergometer until exhaustion. After 10 min of acclimatization in the treadmill chamber the test uptake started at 0.15 m/s, increasing every 3 min by 0.05 m/s. The protocol for the resistance has recently been published [26]. Isometric strength training was performed by the following experimental setup. The mice gripped with their front and their back paws on a horizontal wire of the metal mesh and the plate was placed in a vertical position. The orientation of the mice was holding head up. Strength training was performed for 5 times/week for 3 minutes and 3 series. Break between each series was 1 minute. It was previously demonstrated that this type of isometric strength training was followed by an increase of maximum holding time in isometric strength test, a type II fiber hypertrophy, and an increased GLUT4 protein content in the membrane fraction [26].

### 2.3. Glucose Tolerance Test (GTT)

Glucose tolerance tests were performed at the end of the 10-week experimental period as follows: after an overnight 12-hour fast, blood was drawn from the tail vein of all animals (approximately 10 *μ*L) and glucose concentration measured (glucometer, Roche Diagnostics, Mannheim, Germany). The last training occurred three days before GTT was performed. This was designated as time point zero of the GTT. Glucose (2 g/kg) was applied to the mice via intraperitoneal injection [27]. Blood glucose measurements were repeated after 30, 60, and 120 min.

### 2.4. Tissue Samples and Sample Preparation

Mice were euthanized by 5% isoflurane anesthesia followed by cervical dislocation. The animals were in the fed state when they were euthanized. Blood was collected in EDTA-coated tubes (Sarstedt, Germany) by cardiac puncture with heparin solution-washed 27G syringes (Sarstedt, Germany) and centrifuged for 10 min at 4°C and 1200 ×g. Plasma was stored at −80°C until further analysis. AT surrounding mesenteric lymph nodes was removed immediately after euthanasia and stored at −80°C. We used real-time quantitative PCR to determine TNF-*α*, NLRP-3, IL-1*β*, and IL-18 in isolated AT. The plasma was used for a multiplex ELISA approach (Mouse Inflammation MAP, Myriad RBM, Germany) as well as for determining circulating total fatty acids and ceramides. Serum was tested for the following cytokines: CD40 (CD40), CD40 Ligand (CD40-L), C-reactive protein (CRP), endothelin-1 (ET-1), eotaxin, epidermal growth factor mouse (EGF mouse), factor VII, fibrinogen, fibroblast growth factor 9 (FGF-9), fibroblast growth factor basic (FGF-basic), granulocyte chemotactic protein-2 mouse (GCP-2 mouse), macrophage colony-stimulating factor (M-CSF), growth-regulated alpha protein (KC/GRO), haptoglobin, immunoglobulin A (IgA), interferon gamma (IFN-gamma), interferon gamma-induced protein 10 (IP-10), interleukin- (IL-) 1 alpha (IL-1 alpha), IL-1 beta, IL-10, IL-11, IL-12 subunit p70 (IL-12p70), IL-17A, L-18, IL-2, IL-3, IL-4, IL-5, IL-6, IL-7, leukemia inhibitory factor (LIF), lymphotactin, macrophage colony-stimulating factor-1 (M-CSF-1), macrophage inflammatory protein-1 alpha (MIP-1 alpha), macrophage inflammatory protein-1 beta (MIP-1 beta), macrophage inflammatory protein-1 gamma (MIP-1 gamma), macrophage inflammatory protein-2 (MIP-2), macrophage inflammatory protein-3 beta (MIP-3 beta), macrophage-derived chemokine (MDC), matrix metalloproteinase 9 (MMP9), monocyte chemotactic protein-1 (MCP-1), monocyte chemotactic protein 3 (MCP-3), monocyte chemotactic protein-5 (MCP-5), myeloperoxidase (MPO), myoglobin, oncostatin-M (OSM), serum amyloid P-component (SAP), serum glutamic oxaloacetic transaminase (SGOT), stem cell factor (SCF), T-cell-specific protein, RANTES, thrombopoietin (TPO), tissue factor (TF), tissue inhibitor of metalloproteinase 1 mouse (Timp-1 mouse), tumor necrosis factor alpha (TNF-alpha), vascular cell adhesion molecule-1 (VCAM-1), vascular endothelial growth factor A (VEGF-A), and von Willebrand factor (vWF) by a multiplexed fluorescent bead-based immunoassay (Luminex, Myriad RBM, Austin, Texas).

### 2.5. RNA Isolation, cDNA Synthesis, and Quantitative Real-Time PCR of Adipose Tissue

Adipose tissue (AT) was isolated from mice (for groups see above) and immediately frozen with liquid nitrogen and stored at −80°C until use. RNA was isolated by a combination of Trifast (Peqlab, Erlangen, Germany) and RNeasy Mini Kit (Qiagen, Hilden, Germany). Respective tissue was homogenized with a Precellys 24 (Peqlab, Erlangen, Germany) homogenizer in the presence of Trifast, followed by 5 min incubation on ice and centrifugation at 4°C and 12000 ×g for 10 min. The supernatant was mixed with 200 *μ*L chloroform, followed by 10 min incubation at room temperature. After centrifugation at full speed for 15 min, the upper phase was mixed with an equal amount of 70% ethanol. The mix was loaded on an RNeasy Mini spin column and RNA isolated according to the manufacturer's instructions. The RNA concentrations were measured with a NanoDrop ND-1000 (Kisker-Biotech, Steinfurt, Germany). The isolated RNA (500 ng, each) was converted to cDNA using the iScript cDNA Synthesis Kit (BioRad, Munich, Germany). The conditions for the reverse transcription were as follows: 1 cycle at 25°C for 5 min; 1 cycle at 42°C for 30 min; 1 cycle at 85°C for 5 min. Relative quantification of IL-1*β*, TNF-*α*, NLRP-3, and IL-18 was performed by quantitative real-time PCR with the iQ SYBR Green Supermix in duplets according to the manufacturer's instructions (BioRad, Munich, Germany). Per reaction, a 25 *μ*L mixture was used containing 12.5 *μ*L iQ SYBR Green Supermix, 0.5 *μ*L forward and reverse primer (RPL32-F: 5′-TGGAGGTGCTGCTGATGTG-3′; RPL32-R: 5′-GCGTTGGGATTGGTGACTCT-3′ (product size: 127 bp); IL-1*β*-F: 5′-TGGTGTGTGACGTTCCCATT-3′; IL-1*β*-: 5′-TCGTTGCTTGGTTCTCCTTG-3′ (product size: 172 bp); NLRP-3-F: 5′-TCCAGCACCCAGGCTGTAAC-3′; NLRP-3-R: 5′-TGCAGAGCAGGTGCTTCAGT-3′ (product size: 189 bp); TNF-*α*-F: 5′-AGGGCTGTGGGACCTAAATGT-3′; TNF-*α*-R: 5′-ATGGGATGAGTATGGGGCAGC-3′ (product size: 199 bp); IL-18-F: 5′-GCCGACTTCACTGTACAACCG-3′; IL-18-R: 5′-GAGGGTCACAGCCAGTCCTC-3′ (product size: 182 bp)), 9.5 *μ*L sterile water, and 2 *μ*L of the 1 : 5 diluted complementary DNA template. A nontemplate control was performed in each run. The conditions of the real-time PCR (Mx3000P, Stratagene, Heidelberg, Germany) were as follows: 1 cycle at 95°C for 10 min and then 40 cycles at 95°C for 10 s, 59°C for 10 s, and 72°C for 10 s, followed by a dissociation curve. The intron-spanning primers were designed by using sequence information from the NCBI database. The threshold cycle (Ct) values were normalized to the endogenous control (ribosomal protein L32, RPL32), *n* = 3-4. The specificity of the primer pair products was tested by melting curves and agarose (1.6%) gel electrophoresis.

### 2.6. RNA Isolation, cDNA Synthesis, and Quantitative Real-Time PCR of Liver Tissue

The expression of the following enzymes was investigated: ceramide synthase 2 (CerS2), ceramide synthase 4 (CerS4), fatty acid elongase 1 (ELOVL1), fatty acid elongase 3 (ELOVL3), alkaline ceramidase 2 (ACER2), serine palmitoyltransferase, long chain base subunit 2 (SPTLC2), sphingomyelin phosphodiesterase 1 acid lysosomal (SMPD1), and stearoyl-CoA desaturase (delta-9-desaturase, SCD1). RNA isolation, cDNA synthesis, and qPCR analysis were performed as described recently in detail [28]. In brief, total RNA was isolated from 10 mg liver aliquots using TRIzol reagent (Invitrogen, Karlsruhe, Germany), and RNA concentration and purity were estimated from the optical density at 260 and 280 nm (Infinite 200M microplate reader, Tecan, Männedorf, Switzerland). RNA integrity was assessed by confirming intact bands corresponding to the 18S and 28S ribosomal RNA subunits using 1% agarose gel electrophoresis. Following cDNA synthesis within one week after RNA isolation using dT18 primer and M-MuLV Reverse Transcriptase (MBI Fermentas, St. Leon-Rot, Germany), qPCR runs were performed with a Rotorgene 2000 system (Corbett Research, Mortlake, Australia) as described recently in detail [28]. The used primers were as follows: ceramide synthase 2-F: CAAAGCTGGACCAAGGTTCC, ceramide synthase 2-R: GAGAGGGAGGCAGTGAGATC, ceramide synthase 4-F: CTGTGGGTGTCCTTGTAGTCC, ceramide synthase 4-R: TCCTCTGGCTTTGGTTTCTG, fatty acid elongase 1-F: TCCCATCCCTTCTCCCAGCC, fatty acid elongase 1-R: CCAGCCCTTTCAGCCTCCAG, fatty acid elongase 3-F: AGACCTACCGGCTGTCCTCC, fatty acid elongase 3-R: GAACCGAGTGAGCGTCCAGG, alkaline ceramidase 2-F: AGCCCGCCATCAACAATA, alkaline ceramidase 2-R: CCAGCAGAAGAGAGCCAGAG, serine palmitoyltransferase, long chain base subunit 2-F: CCTGTCAGCAGCTCATACCA, serine palmitoyltransferase, long chain base subunit 2-R: CACACTGTCCTGGGAGGAAT, sphingomyelin phosphodiesterase 1, acid lysosomal-F: GCCAGTCAGCCGTCCATC, sphingomyelin phosphodiesterase 1, acid lysosomal-R: CCCAGAAGCAAGCCACAAG, stearoyl-CoA desaturase (delta-9-desaturase)-F: CGTGGCTTCTTCTTCTCTCTCA, and stearoyl-CoA desaturase (delta-9-desaturase)-R: CTTCTCGGCTTTCAGGTCAG. All primer pairs were designed to have melting temperatures of about 60°C, and if possible, both primers of a primer pair were designed to be located in different exons. Ct values of target genes and reference genes were obtained using Rotorgene Software 5.0 (Corbett Research). For determination of relative expression levels relative quantities were calculated using GeNorm normalization factor. In order to calculate the normalization factor, all Ct values were transformed into relative quantification data by using the 2^−ΔCt^ equation, and the highest relative quantities for each gene were set to 1. From these values the normalization factor was calculated as the geometric mean of expression data of the three most stable out of six tested potential reference genes (ATP5B, CANX, CYC1, EIF4, GAPDH, and RPS9). Reference gene stability across samples from each tissue and each cell line was determined by performing GeNorm analysis [29]. After normalization of gene expression data using the calculated GeNorm normalization factor, means and SD were calculated from normalized expression data for samples of the same treatment group.

### 2.7. Measurement of Circulating Fatty Acids and Ceramides

Lipids were quantified by direct flow injection electrospray ionization tandem mass spectrometry (ESI-MS/MS) in positive ion mode using the analytical setup and strategy described previously [30]. In brief, plasma samples were extracted according to the method by Bligh and Dyer [31] in the presence of nonnaturally occurring lipid species used as internal standards. A fragment ion of* m/z* 264 was applied for sphingosine-based ceramides (Cer) and hexosylceramides (HexCer) with N-heptadecanoyl-sphingosine as internal standard [32]. Fatty acid methyl ester was formed by methanolic acetyl-chloride derivatization and extracted with hexane. Total fatty acid (FA) analysis was carried out by gas chromatography coupled to mass spectrometry (GC-MS) [33].

### 2.8. Statistical Analysis

Two-way ANOVAs with Bonferroni post hoc testing were performed in order to analyze differences between treatments. For the analysis of FA and ceramides a Bonferroni correction was applied with significance levels of *p* < 0.002 (fatty acids) and *p* < 0.005 (ceramides). We present the proinflammatory panel measured by multiplex ELISA as a heat map. To compare the cytokine production between the groups we used a two-way ANOVA with Bonferroni correction and set the new significance cut-off at *p* < 0.001. In order to calculate the corrected *p* value we divided *p* < 0.005 by the number of compared parameters. Furthermore, we calculated percentage changes of the cytokine levels of the HFD groups relative to the mean of the ST/S group. For the analysis of the RT-PCR results from AT, the comparative Ct method was used. Data are shown as 2^(delta)(delta) Ct^ (ΔΔCt) that were calculated by subtracting the mean ΔCt (Ct (reference gene) − Ct (gene of interest)) of control sample from the ΔCt of each examined sample. Two-way ANOVA was used to compare the ΔCt values between the treatments. The statistical analysis of hepatic mRNA expression was performed as a two-way ANOVA on the relative mRNA levels calculated as described above.

## 3. Results

### 3.1. Exercise Reduces Glucose Intolerance and Restricts Weight Gain in HFD Mice

**Figure 1:**
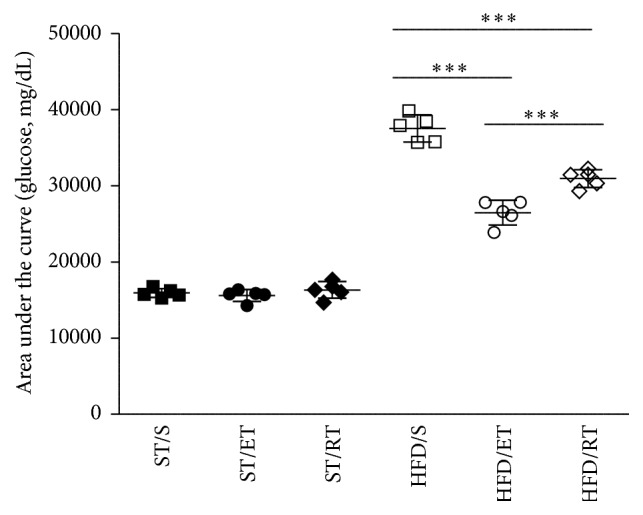
Area under the curve (AUC) of mean blood glucose (mg/dL) of mice fed standard chow or a high fat diet following an intraperitoneal glucose challenge. ST: standard diet, HFD: high fat diet, S: sedentary, ET: endurance training, and RT: resistance training. SD: standard deviation. For each group *n* = 5. ^*∗∗∗*^ *p* < 0.001, comparison within HFD groups: sedentary versus ET or RT and ET versus RT.

**Figure 2:**
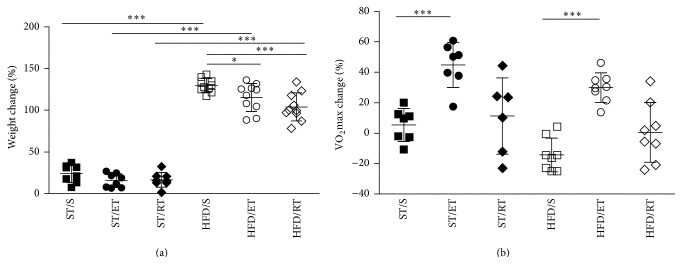
Percentage change in body weight (a) and VO_2_max (b) of untrained and trained mice following either a ST diet or a HFD for 10 weeks. ST: standard diet, HFD: high fat diet, S: sedentary, ET: endurance training, and RT: resistance training. ^*∗∗∗*^ *p* < 0.001, ^*∗*^ *p* < 0.05.

Diet and exercise had significant effects on blood glucose (*p* < 0.01) after glucose challenge. Compared to ST/S group blood glucose levels in the HFD/S group were significantly higher at all-time points after glucose challenge (*p* < 0.001). Both types of exercise led to a significant reduction of glucose intolerance in the HFD animals when compared with the sedentary group. However, the effect was more pronounced after ET (*p* < 0.001; Figure 1). Mice fed the HFD gained significantly more weight than the mice fed the ST diet (*p* < 0.001, Figure 2(a)). Within the high fat group, both ET (*p* < 0.05) and RT (*p* < 0.001) attenuated the weight gain when compared with the sedentary group. VO_2_max change was measured at baseline before exercise commenced as well as after 10 weeks on exercise. The percent changes in VO_2_max for each diet and exercise group are shown in Figure 2(b). ET but not RT led to significant increases of VO_2_max in both ST and HFD groups (*p* < 0.001).

### 3.2. Ceramide Profiles Are Affected by Exercise

The HFD significantly increased most of the total fatty acids and ceramides in plasma of mice from HFD/S group (*p* < 0.005; Tables 2 and 3). 13 out of 19 fatty acids analyzed were increased while 6 were unaffected. Neither ET nor RT was able to reduce significantly any fatty acid compared to the enhanced levels of sedentary mice. Eight out of 10 ceramides analyzed increased significantly in plasma from HFD/S mice compared to ST/S mice (Table 3). ET and RT affected ceramide levels slightly different in HFD mice. While ET decreased Cer d23:0 and d24:1, RT was followed by a significant decrease of Cer d24:1 and HexCer d24:1 (*p* < 0.005; Table 3).

### 3.3. Exercise Reduces Hepatic Expression of CerS4 and SPTLC2 in HFD Mice

Endurance training significantly decreased the relative mRNA levels of CerS2, ELOVL1, and ELOVL3 as well as SMPD1 (*p* < 0.05) in comparison with the sedentary ST group (Figures 5(a), 5(c), 5(d), and 5(g)). Furthermore, ET increased the relative mRNA level of CerS2 (*p* < 0.05) in the HFD in comparison to the corresponding ST fed mice. In contrast, ELOVL3 was strongly reduced in the ET group due to the HFD feeding. RT had no effect on CerS2 and ELOVL1 expression. However, ELOVL3 was reduced by RT only in the ST group (Figures 5(c) and 5(d)). ELOVL1 and ELOVL3 mRNA expression decreased in the sedentary group due to the high fat diet. SCD1 and ACER2 were not significantly changed in the ST groups. However, we observed a trend towards a reduction of mRNA expression due to exercise in the HFD groups that did not reach statistical significance (Figures 5(e) and 5(h)). The comparison within the HFD groups revealed that ET significantly reduced CerS4 and SPTLC2 mRNA. Resistance training had a similar effect on SPTLC2 but did not reach significance for CerS4 (Figure 5(f)).

### 3.4. Exercise Reverses HFD-Induced Alterations of Proinflammatory Cytokines

**Figure 3:**
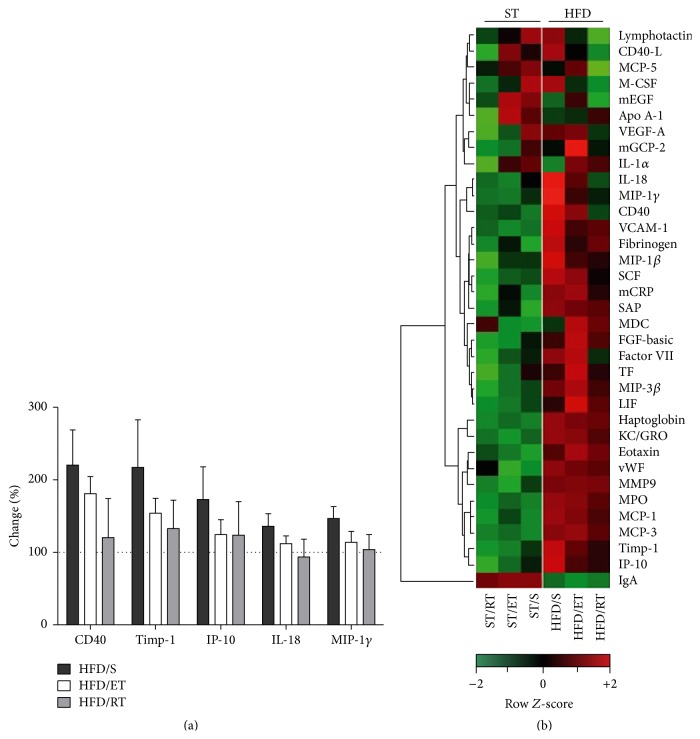
(a) Percent change in cytokine levels in the high fat groups relative to the ST/S group. Exercise, in particular resistance training, showed the tendency to partially reverse the HFD-induced increase in several cytokines. For each individual animal the percentage change in cytokine level relative to the mean cytokine levels of the ST/S group was calculated. The black bars represent the percentage changes in cytokine production after HFD/S in comparison to the mean of the ST/S group. (b) Heat map summarizing cytokine production in plasma samples of mice fed either a ST diet or a HFD that were subjected to endurance and resistance training, as well as sedentarism. Each column shows the mean of six animals per group. The scale represents the *Z*-scores from the log2-transformed values of each cytokine (red indicates *Z* > 0, green indicates *Z* < 0, and black indicates *Z* = 0). ST: standard diet, HFD: high fat diet, S: sedentary, ET: endurance training, and RT: resistance training. For each group *n* = 6. MCP-5: monocyte chemotactic protein-5, M-CSF: macrophage colony-stimulating factor-1, mEGF: epidermal growth factor mouse, Apo A-I: apolipoprotein A-I, VEGF-A: vascular endothelial growth factor A, mGCP-2: granulocyte chemotactic protein-2 mouse, IL-1*α*: interleukin 1 alpha, MIP-1*β*: macrophage inflammatory protein-1*β*, VCAM-1: vascular cell adhesion molecule-1, MIP-1*γ*: macrophage inflammatory protein-1 gamma, SCF: stem cell factor, mCRP: C-reactive protein mouse, SAP: plasma amyloid P-component, MDC: macrophage-derived chemokine, FGF-basic: fibroblast growth factor basic, TF: tissue factor, KC/GRO: growth-regulated *α* protein, vWF: von Willebrand factor, MMP9: matrix metalloproteinase 9, MPO: myeloperoxidase, MCP-3: monocyte chemotactic protein 3, Timp-1: tissue inhibitor of metalloproteinase 1 mouse, and IP-10: interferon gamma-induced protein 10.

After exposure to HFD a number of cytokines increased in sedentary animals. Plasma concentrations of eotaxin, haptoglobin, MCP-1, MCP-3, MPO, CD40, KC/GRO, SAP, VCAM-1, vWF, Timp-1, IP-10, IL-18, and MIP-1*γ* were significantly increased in HFD/S group compared to the ST/S (*p* < 0.001). Alterations of some cytokines could be reversed by exercise intervention. Again, exercise regimes differently affect cytokine expression in the HFD groups (Figure 3(b)). While ET significantly reduced cytokine levels of MIP-1*γ*, RT was followed by a significant reduction of CD40, IL-18, MIP-1*γ*, and Timp-1 (*p* < 0.001). In addition, we show in Figure 3(a), as a supplementary graph, the percentage change of selected cytokines levels after both ET and RT in the HFD groups which represent the most prominent effects. This should illustrate the effect of exercise on the cytokine level of high fat diet fed mice. Notably, mice fed the HFD showed a decrease of plasma immunoglobulin A (*p* < 0.001) which was not affected by any exercise intervention.

### 3.5. Exercise Differently Downregulates the Expression of Inflammatory Markers in Adipose Tissue

**Figure 4:**
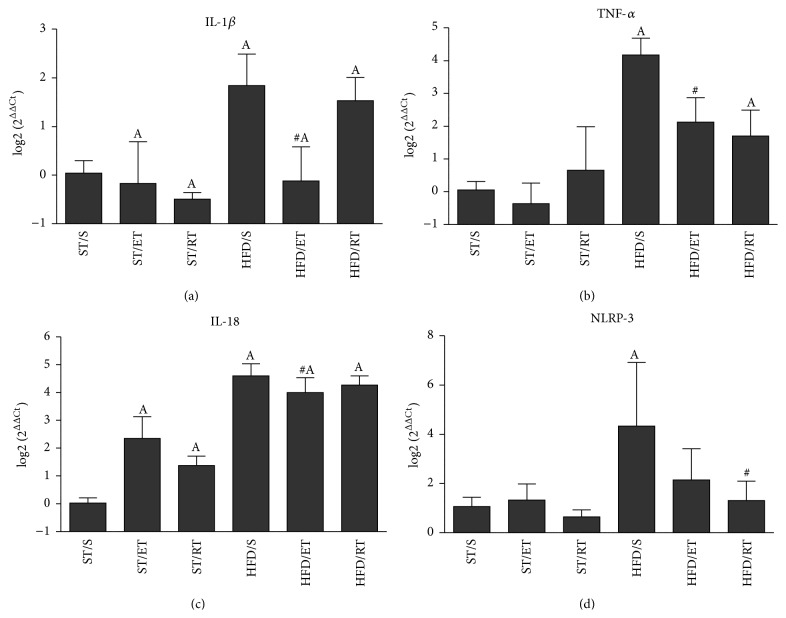
Adipose tissue mRNA expression of IL-1*β* (a), TNF-*α* (b), IL-18 (c), and NLRP-3 (d) in sedentary and exercised groups of animals fed a standard or a high fat diet. Delta delta Ct (ΔΔCt) values were calculated by subtracting the mean ΔCt of ST/S (control) from the ΔCt of each examined sample. Statistical comparison was performed on ΔCt of each group. Data are shown as log2 (2^ΔΔCt^) values. A: comparison between ST diet groups and corresponding HFD group (*p* < 0.05), #: comparison between sedentary HFD group and the exercised HF groups, ST: standard diet, HFD: high fat diet, S: sedentary, ET: endurance training, and RT: resistance training.

**Figure 5:**
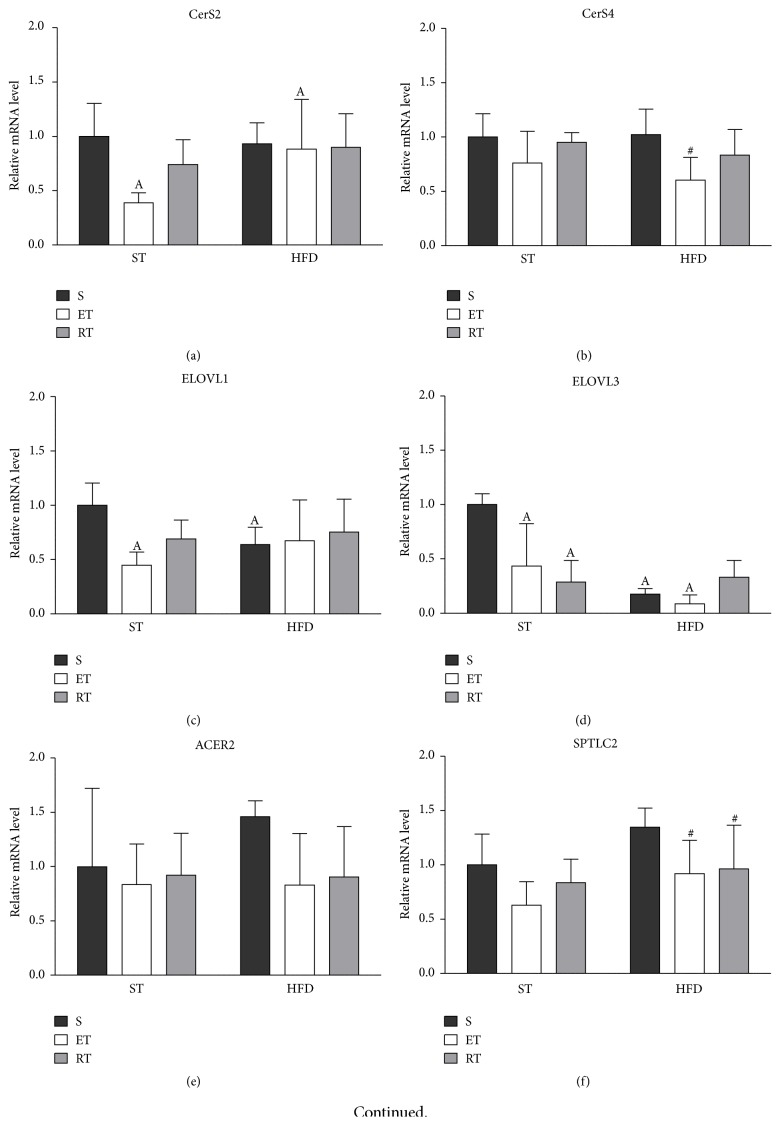
Relative mRNA of genes involved in ceramide biosynthesis pathway in liver of exercise HFD and ST fed mice. A detailed description of the calculation of the relative mRNA levels can be found in Material and Methods. Briefly, all Ct values were transformed into relative quantification data by using the 2^−ΔCt^ equation, and the highest relative quantities for each gene were set to 1. (a) CerS2: ceramide synthase 2, (b) CerS4: ceramide synthase 4, (c) ELOVL1: fatty acid elongase 1, (d) ELOVL4: fatty acid elongase 3, ACER2: alkaline ceramidase 2, (f) SPTLC2: serine palmitoyltransferase, long chain base subunit 2, (g) SMPD1: sphingomyelin phosphodiesterase 1 (acid lysosomal), and (h) SCD1: stearoyl-CoA desaturase-1. HFD: high fat diet, ST: standard diet, S: sedentary, ET: endurance training, and RT: resistance training. A: comparison between ST diet groups and corresponding HFD group (*p* < 0.05); #: comparison between sedentary HFD group and the exercised HF groups.

Feeding the HFD was followed by a significant increase of IL-18, TNF-*α*, and NLRP-3 mRNA expression in AT, while the enhanced mRNA expression of IL-1*β* did not reach significance (Figures 4(a)–4(d)). The two types of exercise intervention affected the inflammatory markers differently. ET was accompanied by a significant reduced expression of TNF-*α* and IL-1*β* compared to HFD/S group. In contrast, RT significantly reduced only the NLRP-3 expression (Figure 4(d)). A secondary finding was that both exercise treatments were effective in enhancing IL-18 expression in AT of ST fed mice.

## 4. Discussion 

We showed that HFD-induced obesity was associated with a substantial increase in body weight, a decrease of physical performance, elevated levels of serum ceramides and fatty acids, both a local, in AT, and a systemic inflammatory response, and finally glucose intolerance. Both training interventions, endurance as well as resistance exercise, were effective in reversing or in attenuating the diet-induced glucose intolerance. However ET reduced blood glucose more efficiently than RT (*p* < 0.001) probably due to higher energy expenditure in the ET group. It is speculated that the exercise effect might be mediated via metabolic and inflammatory pathways as indicated by decreased levels of serum ceramides and reduced levels of tissue and serum inflammatory markers.

Obesity-associated tissue inflammation is assumed to be an important factor in the activation of the innate immune system leading to a systemic chronic low grade inflammation. It has been suggested that the starting point for AT inflammation might be the cell stress-associated accumulation of metabolites which have been shown to dramatically increase in response to HFD feeding [34]. In this regard, ceramides may represent a potential upstream initiating event for obesity-induced inflammation [18]. Current data demonstrated that high fat feeding induced an increase of several ceramides in serum, an observation that is in line with previous studies [19]. Likewise, ceramide transported in LDL has been shown to be elevated in the plasma of obese patients with type 2 diabetes [35]. Incubating cultured myotubes with reconstituted low density lipoprotein ceramide 24:0 promoted ceramide accrual in cells and was accompanied by reduced insulin-stimulated glucose uptake. Moreover, in lean mice the infusion of LDL-ceramide reduced insulin-stimulated glucose uptake, and this was due to impaired insulin action specifically in skeletal muscle [35]. Ceramide seems to induce insulin resistance by inhibiting insulin signal transduction, principally at Akt [36]. Ceramide is also postulated to activate proinflammatory pathways in macrophages, perhaps via amplification of TLR 4-mediated inflammation [18]. Therefore, it has been suggested that changes in plasma long chain ceramides might link the effects of HFD on the inflammatory status of adipose tissue. We did not determine macrophage phenotype in adipose tissue but showed that an increased expression of TNF-*α*, IL-18, and NLRP-3 which was detected in adipose tissue of HFD/S group might indicate adipose tissue inflammation. These findings are in line with previous studies which found an increased inflammatory status in AT in response to chronic high fat feeding [34, 37]. Interestingly, both exercise regimes differently affected expression of inflammatory markers in AT. While ET significantly reduced the expression of TNF-*α*, IL-1*β*, and IL-18, RT reduced NLRP-3 expression. Thereby, a reduced expression of TNF-*α* and IL-1*β* might be interpreted as a reduction of macrophage and lymphocyte activation [10]. Although we did not determine caspase activity, the reduced expression of IL-1*β* might represent a decrease in the maturation of IL-1*β* by a decrease in NLRP-3 dependent caspase 1 activity [18, 38]. However, a reduction of caspase 1 activity has been shown to be linked with lower levels of ceramides in serum, which has been demonstrated in our study after the exercise interventions [38]. Therefore, our study suggests that a starting point for the anti-inflammatory effects of exercise training might be the reduction in ceramide levels. These results are in line with previously published research that demonstrated the essential involvement of ceramides in the development of insulin resistance [39, 40]. Exercise significantly decreased Cer d18:1/23:0 and d18:1/24:1 in the HFD/ET group and d18:1/24:1 and HexCer d18:1/24:1 in the HFD/RT group when compared to the HFD/S group. The ceramides measured are secreted by the liver and bound to lipoproteins. It has been shown that Cer 24:1 and 24:0 make up about 80% of all measured plasma ceramides. Ceramides like reconstituted low density lipoprotein ceramide 24:0 were found to increase TNF-*α*, IL-1*β*, IL-6, and MCP-1* in vitro* and to induce insulin resistance in lean mice [35]. It is therefore assumed that the exercise-induced reduction of plasma long chain ceramides is at least one reason for the reduced expression of inflammatory markers in serum and AT as well as the increased glucose tolerance.

Because of the reduced levels of ceramides in plasma, we measured whether exercise affects the expression of genes involved in the synthesis of ceramides. CerS2 and ELOVL1 have been shown to be essential for the synthesis of Cer d24:0 and Cer d24:1 [41]. The hepatic mRNA expression levels of CerS2 and CerS4 do not change significantly in the sedentary HFD mice groups which might suggest adequate substrate availability under condition of our HFD protocol. ELOVL1 and ELOVL3 decreased significantly in the HFD/S group in comparison with the ST/S group. Since the HFD feeding led to a significant increase in long chain FAs, this downregulation of elongases might indicate a feedback loop mechanism where a high pool of FA downregulates mRNA expression. In murine hepatocytes, the expression of CerS4 is far below that of CerS2, which is the most abundantly expressed ceramide synthase in mammals. CerS2 and CerS4 share some substrate specificity; for example, CerS4 utilizes C18-C22 FA, whereas CerS2 utilizes C20-C26 FA [42, 43]. Furthermore, the synthesis rate of CerS* in vitro* has been shown to be dose-dependent and that ceramide synthesis is governed by the availability of FA [44, 45]. In addition, our results showed that SPTLC2 expression was significantly reduced by exercise which might also point towards a decline of available FA for ceramide synthesis. Therefore, we can speculate that exercise affects blood ceramide profile mostly through an alteration of substrate availability for ceramide synthesis rather than a change in expression of gene involved in the ceramide pathway.

Another reason for the reduced expression of inflammatory markers in serum and AT might be the effects of both exercise and diet on the expression of IL-18. IL-18 is generally considered a proinflammatory cytokine, associated with obesity and insulin resistance. Alterations in IL-18 might have been responsible for both the downregulation of glucose tolerance in HFD/S group and the increased glucose sensitivity after exercise training. These assumptions are supported by previous studies which found that obese individuals had higher IL-18 mRNA content in the abdominal adipose tissue than nonobese subjects and that exercise training lowered the elevated IL-18 mRNA levels in obese subjects [46]. Activation of the NLRP-3 inflammasome in course of diet-induced obesity in mice led to an increase in IL-18 secretion and was shown to affect insulin signaling [18]. In addition, it has been shown that IL-18 mRNA and plasma IL-18 correlated with insulin resistance, suggesting that training-induced reduction in IL-18 expression may be a contributing mechanism to improve insulin sensitivity after training [18, 46, 47].

We found that IL-18 mRNA expression in AT was increased in all high fat groups but was not changed by exercise. The levels of plasma IL-18, however, were significantly increased in the HFD/S group and were reduced by endurance as well as by resistance training. This was associated with improved glucose tolerance in the exercised groups [46]. The results of our study therefore suggest that the reduction of NLRP-3 levels in AT was not effective enough in reducing IL-18 mRNA expression in AT. However, we speculate that it affected posttranslational modification leading to a reduced maturation of IL-18 protein in AT or a reduced mobilization of IL-18 into the blood stream. The role of IL-18 during development of obesity seems to be ambivalent as it has been shown that deficiency of either IL-18 or IL-18 receptor triggers obesity and hyperinsulinemia in mice and that treatment with recombinant IL-18 reversed these effects [47]. Additionally, IL-18 elevated adenosine monophosphate-activated protein kinase in skeletal muscle which is known to increase energy expenditure and endurance in mice [48, 49]. These studies indicate that IL-18 might play an essential role in metabolic regulation and exercise adaptation. In the current study the expression of IL-18, in AT, was significantly increased in the exercised standard fed mice when compared with the ST/S animals. Additionally, we did not detect elevated glucose levels in the exercising ST groups. These results suggest that the role of IL-18 in AT is that of a metabolic key cytokine which might also be involved in the adaptation to exercise.

In addition to IL-18, exercise was effective in reducing TNF-*α* mRNA expression which might represent another pathway of action. Regarding the connection between inflammation and insulin resistance, it is known that TNF-*α* induces insulin resistance* in vitro* and in mice [50]. However, in contrast to IL-18 a decrease of TNF-*α* was found in AT after exercise while no changes of peripheral TNF-*α* were detected.

The localized inflammatory environment inside the adipose tissue seemed to have systemic consequences as reflected by enhanced levels of several proinflammatory serum cytokines. A cluster of proinflammatory cytokines increased in the HFD/S group might indicate a state of chronic low grade inflammation [17]. Thereby, notable increases of CD40 might be the result of an activation of lymphocytes, while cytokines like IL-18, MIP-1*γ*, and Timp-1 might point towards an increased activation of macrophages [8]. Both exercise types reversed the diet-induced increase of some of these cytokines. While RT mainly affected CD40, IL-18, MIP-1*γ*, and Timp-1 expression, ET effectively reduced MIP-1*γ*. Since both exercise types reduced body weight, we cannot exclude the possible confounding effect of this weight loss and potential lower body fat content on the reduction of inflammatory markers. A major limitation of this study is that our HFD model contained 34% lipids by weight, which is a moderate challenge. However, we propose that exercise diminishes both lymphocyte and macrophage activity which is in accordance with previously published data [10].

Taken together, we show that HFD-induced obesity is accompanied by an increase of ceramides, activation of inflammasome in AT, and a secretion of several proinflammatory cytokines including IL-18 that all are known to affect glucose sensitivity. Both ET and RT are effective therapeutic strategies to improve both metabolic dysfunction and inflammatory status resulting in an improved glucose tolerance. However, the identification of definitive molecular targets for both training regimes, which seem to address slightly different signaling pathways, has to be elucidated in future studies.

## Figures and Tables

**Table 1:** Fatty acid composition of standard (low fat) diet and high fat diet.

Fatty acid	ST	HFD
	mg/kg	mg/kg
10:0	—	350
12:0	—	350
14:0	—	5250
16:0	2236	64580
16:1	—	6650
17:0	—	1400
18:0	688	63000
18:1 (n-9)	3956	127750
18:2 (n-6)	9366	39550
18:3 (n-3)	1282	3150
20:0	—	1050
20:1 (n-9)	—	4200
20:2 (n-6)	—	1750
20:4 (n-6)	—	700

ST: standard diet; HFD: high fat diet. —: not added.

**Table 2:** Effects of exercise on the plasma total fatty acid profiles of standard diet or HFD mice.

Fatty acid	ST/S	ST/ET	ST/RT	HFD/S	HFD/ET	HFD/RT
(*μ*mol/L)	Mean (SD)	Mean (SD)	Mean (SD)	Mean (SD)	Mean (SD)	Mean (SD)
12:00	6.0 (0.50)	6.0 (0.50)	6.0 (0.30)	7.0 (2.00)	8.0 (2.00)^a^	8.0 (1.00)^a^
14:00	22.0 (3.00)	21.0 (2.00)	26.0 (3.00)	38.0 (7.00)^a^	46.0 (17.00)^a^	47.0 (10.00)^a^
15:00	9.0 (2.00)	9.0 (1.00)	11.0 (1.00)	6.0 (1.00)	7.0 (1.00)	7.0 (0.40)
16:00	1657000.0 (254.00)	1461000.0 (147.00)	1777000.0 (116.00)	2200.0 (431.00)^a^	2200.0 (281.00)^a^	2149000.0 (145.00)
16:01	254.0 (56.00)	224.0 (37.00)	279.0 (36.00)	176.0 (27.00)^a^	179.0 (42.00)	169.0 (14.00)^a^
17:00	12.0 (2.00)	11.0 (1.00)	13.0 (0.40)	17.0 (2.00)^a^	18.0 (2.00)^a^	18.0 (1.00)^a^
18:00	437.0 (49.00)	373.0 (31.00)	435.0 (14.00)	1368.0 (260.00)^a^	1400.0 (91.00)^a^	1374.0 (70.00)^a^
18:1 (n-9)	1174000.0 (177.00)	1081000.0 (141.00)	1333000.0 (91.00)	1669.0 (133.00)^a^	1711.0 (88.00)^a^	1694000.0 (221.00)
18:1 (n-7)	167.0 (17.00)	162.0 (18.00)	182.0 (11.00)	128.0 (14.00)^a^	133.0 (19.00)	121.0 (16.00)^a^
18:2 (n-6)	1664000.0 (269.00)	1494000.0 (132.00)	1811000.0 (66.00)	2290.0 (486.00)^a^	2318.0 (281.00)^a^	2328.0 (95.00)^a^
18:3 (n-3)	18.0 (5.00)	20.0 (2.00)	26.0 (3.00)	17.0 (4.00)	20.0 (8.00)	21.0 (5.00)
20:00	7.0 (2.00)	6.0 (1.00)	8.0 (1.00)	9.0 (2.00)	10.0 (3.00)	11.0 (2.00)
20:1 (n-9)	28.0 (11.00)	21.0 (2.00)	26.0 (3.00)	27.0 (7.00)	26.0 (6.00)	38.0 (9.00)
20:3 (n-6)	79.0 (5.00)	64.0 (6.00)	72.0 (9.00)	157.0 (62.00)^a^	147.0 (33.00)^a^	119.0 (32.00)
20:4 (n-6)	665.0 (47.00)	516.0 (80.00)	573.0 (74.00)	2005.0 (780.00)^a^	2143.0 (270.00)^a^	1720.0 (297.00)^a^
20:5 (n-3)	9.0 (2.00)	8.0 (1.00)	10.0 (1.00)	30.0 (10.00)^a^	36.0 (2.00)^a^	31.0 (4.00)^a^
22:4 (n-6)	11.0 (1.00)	9.0 (1.00)	11.0 (1.00)	15.0 (4.00)	15.0 (2.00)^a^	14.0 (1.00)
22:5 (n-3)	10.0 (1.00)	9.0 (1.00)	10.0 (2.00)	17.0 (5.00)^a^	19.0 (3.00)^a^	19.0 (3.00)^a^
22:6 (n-3)	162.0 (5.00)	121.0 (13.00)	137.0 (13.00)	576.0 (222.00)^a^	594.0 (47.00)^a^	513.0 (116.00)^a^

ST: standard diet, HFD: high fat diet, S: sedentary, ET: endurance training, and RT: resistance training. SD: standard deviation. For each group *n* = 5. ^a^ST fed groups versus corresponding HFD groups (*p* < 0.002).

**Table 3:** Ceramide profiles of mice following a standard diet or HFD fed mice for 14 weeks.

Ceramide	ST/S	ST/ET	ST/RT	HFD/S	HFD/ET	HFD/RT
(*μ*mol/L)	Mean (SD)	Mean (SD)	Mean (SD)	Mean (SD)	Mean (SD)	Mean (SD)
d18:1/16:0	0.253 (0.027)	0.228 (0.020)	0.255 (0.044)	0.749 (0.182)^a^	0.723 (0.097)^a^	0.659 (0.119)^a^
d18:1/18:0	0.039 (0.027)	0.051 (0.029)	0.057 (0.027)	0.192 (0.096)^a^	0.201 (0.058)^a^	0.174 (0.059)^a^
d18:1/20:0	0.079 (0.004)	0.062 (0.026)	0.073 (0.019)	0.483 (0.202)^a^	0.398 (0.113)^a^	0.439 (0.145)^a^
d18:1/22:0	0.677 (0.089)	0.477 (0.118)	0.576 (0.103)	2.672 (1.120)^a^	2.060 (0.286)	2.404 (1.001)^a^
d18:1/23:0	0.350 (0.046)	0.373 (0.070)	0.397 (0.033)	0.627 (0.149)^a^	0.462 (0.052)^*∗*^	0.521 (0.095)
d18:1/24:1	1.722 (0.065)	1.261 (0.097)	1.323 (0.108)	2.782 (0.939)	1.815 (0.376)^*∗*^	1.819 (0.284)^*∗*^
d18:1/24:0	1.027 (0.185)	0.819 (0.119)	0.949 (0.142)	1.035 (0.163)	0.814 (0.083)	0.950 (0.155)
HexCer d18:1/16:0	0.274 (0.062)	0.239 (0.030)	0.252 (0.019)	0.860 (0.197)^a^	0.791 (0.142)^a^	0.725 (0.093)^a^
HexCer d18:1/24:1	0.815 (0.126)	0.698 (0.063)	0.806 (0.082)	1.466 (0.307)^a^	1.398 (0.223)^a^	1.092 (0.155)^*∗*^

ST: standard diet, HFD: high fat diet, S: sedentary, ET: endurance training, and RT: resistance training. SD: standard deviation. For each group *n* = 5.  ^a^ST fed groups versus corresponding HFD groups (*p* < 0.005), ^*∗*^comparison within ST groups or HFD groups, sedentary versus ET or RT (*p* < 0.005): HexCer: hexosylceramide.
